# The “1958 He^4^ Scale of Temperatures”^1^: Part 1. Introduction

**DOI:** 10.6028/jres.064A.001

**Published:** 1960-02-01

**Authors:** F. G. Brickwedde

## Abstract

The generally used practical scale of temperatures between 1° and 5.2° K is the He^4^ vapor pressure scale based on an accepted vapor pressure equation or table. In Sèvres (near Paris), October 1958, the International Committee on Weights and Measures recommended for international use the “1958 He^4^ Scale” based on a vapor pressure table arrived at through international cooperation and agreement. This table resulted from a consideration of all reliable He^4^ vapor pressure data obtained using gas thermometers, and paramagnetic susceptibility and carbon resistor thermometers. The theoretical vapor pressure equation from statistical thermodynamics was used with thermodynamic data on liquid He^4^ and the vapor equation of state to insure satisfactory agreement of the vapor pressure table with reliable thermodynamic data.

The International Committee on Weights and Measures at a meeting in Sèvres (near Paris), France, September 29 to October 3, 1958, approved the “1958 He^4^ Vapor Pressure Scale of Temperatures” as an international standard for thermometry from 1° to 5.2° K. This was the culmination of several years of intensive research and cooperation on the helium vapor pressure scale at the Kamerlingh Onnes Laboratory in Leiden, Holland, and the U.S. Naval Research Laboratory in Washington.

The vapor pressure of liquid He^4^ has for a long time been used as a standard for thermometry between 1° and 5.2° K. The first measurements of thermodynamic temperatures in the liquid He^4^ range were made with constant volume gas thermometers filled with He^4^. Simultaneous measurements of the vapor pressure of liquid helium in temperature equilibrium with the gas thermometer established a vapor pressure-temperature relation which then was used as the basis for determining thermodynamic temperatures from vapor pressure measurements. With these vapor pressure-gas thermometer measurements there were measurements of He^4^ vapor pressures made simultaneously with measurements of the He^4^ isotherms from which temperatures were obtained by extrapolating the isotherms to zero density (*N*/*V*→0) in accordance with the virial equation of state:

$$pV/N=RT[1+B(N/V)+C{\left(N/V\right)}^{2}+\ldots ]$$ (1)

After the latent and specific heats of liquid He^4^ had been measured, the experimental vapor pressure-temperature relation was improved through the use of the theoretical vapor pressure (*P*) equation:

$$\ln\;P=i_{0}-\frac{L_{0}}{RT}+\frac{5}{2}\ln\;T-\frac{1}{RT}\int_{0}^{T}S_{l}\;dT+\frac{1}{RT}\int_{0}^{P}V_{l}dP+\epsilon$$ (2)

where

$$i_{0}\equiv \ln\;{\left(2\pi m\right)}^{3/2}k^{5/2}/h^{3}$$ (3)

and

ϵ≡ln(PV/NRT)−2B(N/V)−(32)C(N/V)2 (4)

*L*_0_ is the heat of vaporization of liquid He^4^ at 0° K, *S_l_* and *V_l_* are the molar entropy and volume of liquid He^4^, *m* is the mass of a He^4^ atom, *B* and *C* are the virial coefficients in eq (1), and the other symbols have their usual meaning. Both theoretically calculated and directly measured vapor pressures were considered in arriving at the 1958 He^4^ Temperature Scale.

Equation (2) presupposes that the thermodynamic properties entering the equation have been measured on the thermodynamic scale, otherwise the use of this equation for the calculation of *P* is not valid. In practice, however, these properties are measured on an empirical scale that only approximates the thermodynamic scale. In general this empirical scale has been a He^4^ vapor pressure scale based on gas thermometer measurements.

As *T* is lowered, the fourth, fifth, and sixth terms in eq (2) become smaller and less important relative to the first three terms. At 1.5° K, the inclusion or exclusion of the sum of the fourth, fifth, and sixth terms in eq (2) affects the temperature calculated from a given value of *P* by only 0.0005 deg. It may be said then, that below 1.5° K, the vapor pressure of He^4^ is in effect really determined, within the present accuracy of the vapor pressure measurement, by a single empirical constant, the heat of vaporization of liquid He^4^ at 0° K. At present, *L*_o_ for He^4^ is normally calculated from vapor pressure data obtained with a gas thermometer. The magnitude of the last three terms in eq (2) increases rather rapidly with rising *T*, and above the λ-point (2.172° K) the accuracy of the evaluation of these terms is a very important consideration.

In Amsterdam in 1948, on the occasion of a General Assembly of the International Union of Physics, a small group of low temperature physicists, meeting informally, agreed to use and recommend for temperature measurements between 1° and 5.2° K, a table of vapor pressures of He^4^, then in use in Leiden, which came to be known as the “1948 Scale” [1].^5^ This scale has sometimes been referred to as the “1949” Scale. From 1° to 1.6°K, the “1948 Scale” was based on vapor pressures calculated by Bleaney and Simon [2] using eq (2). From 1.6° to 5.2° K, the scale was based on measured vapor pressures and temperatures determined with gas thermometers. From 1.6° to 4.2° K, it was based primarily on the vapor pressure measurements of Schmidt and Keesom [3].

Even in 1948, when the “1948 Scale” was agreed to, there was evidence in the measurements and calculations of Kistemaker [4] that the “1948 Scale” deviated significantly from the thermodynamic scale. However, it was thought at the time that, on general principles, indicated changes in an existing scale should be made only after these changes had been confirmed. With improvements in the precision and accuracy of physical measurements at low temperatures, irregularities appeared in the temperature variation of physical properties between 1° and 5° K that were in the main reproducible in different substances and properties and were, therefore, attributable to errors in the “1948 Scale” [5]. Stimulated by these results which corroborated Kistemaker’s work, the investigations of the He^4^ vapor pressure scale were undertaken that culminated in the “1958 He^4^ Scale.”

Paramagnetic susceptibility and carbon resistor thermometers were later employed in investigations of the He^4^ vapor pressure-temperature relation [6]. These thermometers were used for the interpolation of temperatures between calibration points (temperatures) using an assumed relation connecting temperature and paramagnetic susceptibility or carbon resistance for the calculation of the temperatures. For suitably chosen paramagnetic salts, the Curie-Weiss Law was assumed to hold:

$$\chi =\frac{C}{T+\Delta }$$ (5)

where χ is the magnetic susceptibility and *C* and Δ are empirical constants. Measurements at two temperatures would suffice to determine these two empirical constants if the measurement were really of χ or a quantity directly proportional to χ. However, a calibration of the paramagnetic thermometer at a third calibration temperature is necessary because the arbitrariness in the size and arrangement of the paramagnetic salt samples and the induction coils that surround the salt sample for the susceptibility measurement make the measurement a linear function of χ. Interpolation equations for carbon resistor thermometers are not as simple as eq (5) and do not have a theoretical basis. Hence, vapor pressure data obtained with carbon resistor thermometers are of more limited usefulness for the determination of the He^4^ vapor pressure-temperature relation. Clement used carbon thermometer data to examine the derivative *d* (ln *P*)/*d* (1/*T*), [7].

Important use has been made of He^4^ vapor pressure measurements made with magnetic susceptibility and carbon resistor thermometers in arriving at the “1958 He^4^ Scale.” These vapor pressure measurements were considered along with those made with gas thermometers and vapor pressures calculated using eq (2). Temperature measurements with magnetic and carbon resistor thermometers are much simpler to make than measurements with gas thermometers, and hence vapor pressure data obtained with magnetic and carbon resistor thermometers are more numerous. Also, the measurements made with these secondary thermometers are more precise (to be distinguished from accurate) which makes them especially useful for interpolation between the gas thermometer data.

There are, accordingly, three practical methods for determining the He^4^ vapor pressure-temperature relation: (1) By use of the direct vapor pressure measurements made with gas thermometers, (2) through the use of eq (2) with some vapor pressure-gas thermometer data, and (3) through the use of vapor pressure measurements with secondary thermometers which have been calibrated using some gas thermometer data. If all the pertinent experimental data were accurate and all temperatures were on the thermodynamic scale, these three methods would yield results in good agreement with each other, and any one might be relied upon for the construction of the He^4^ vapor pressure-temperature table defining the scale. Because of experimental errors, however, the vapor pressures obtained by the different methods differ when carried to the limit of the sensitivity of the measurements. For He^4^ between 1° and 4.5° K, different choices of the methods and different selections of the experimental data used, weighting factors and corrections to the published data yield scales all within about 4 millidegrees of each other. The primary evidence for this is that 4 millidegrees is the maximum difference between the L55 Scale [8] obtained by method (2) and the 55E Scale [9] obtained by method (3). This then is a measure of the range (total spread) of uncertainty at present in the He^4^ vapor pressure scale of temperatures between 1° and 4.5° K.

All published He^4^ vapor pressure measurements, and thermodynamic data needed for eq (2) were independently studied and correlated by H. Van Dijk and M. Durieux at the Kamerlingh Onnes Laboratory in Leiden [8] and by J. R. Clement and J. K. Logan at the U.S. Naval Research Laboratory in Washington [9]. As far as possible, the experimental data of the original investigators were recalculated on the basis of later knowledge of the temperature scale, fundamental constants, and the properties of He^4^. In some cases, limitations were imposed on these recalculations by the incomplete reporting of the experimental data by the original investigator.

After working independently, van Dijk and Clement cooperated to compromise their differences. They met first in Leiden, August 1955 and later in Washington, summer of 1957. From January 22 to March 14, 1958, Logan worked at Leiden, and later represented Clement at a conference in Leiden, June 1958, at which agreement was reached on the “1958 He^4^ Scale.” This cooperation was an important factor in the improvement of the scale.

Where the differences between the values obtained by handling the experimental data differently are largest (4 millidegrees), the “1958 Scale” falls between the extremes. At other places it is close to the mean of these values and at no place does it deviate by more than 2 millidegrees from the mean. The estimated uncertainty of the “1958 He^4^ Scale” is accordingly ±2 millidegrees between 1° and 4.5° K. At higher temperatures, the estimated uncertainty is larger.

Now that the International Committee on Weights and Measures has recommended the “1958 He^4^ Scale” as an international standard it is presumed that henceforth the International Committee on Weights and Measures will take the initiative in improving the scale when changes are needed. Before the International Committee on Weights and Measures assumed responsibility for the He^4^ vapor pressure scale, the Commission on Very Low Temperature Physics in the International Union of Pure and Applied Physics concerned itself with the scale. This began with the informal meeting in Amsterdam in 1948 that resulted in the “1948 Scale.” At the Low Temperature Conferences sponsored by the Commission on Very Low Temperature Physics of the International Union of Physics at Paris in 1955, and at Madison, Wisconsin, in 1957, sessions were held at which the He^4^ vapor pressure scale of temperatures was discussed.

The National Bureau of Standards sponsored meetings, for discussion of the helium vapor pressure scale of temperatures, held at the NBS during the spring meetings of the American Physical Society in Washington, 1955 and 1957. Also, the NBS encouraged cooperation in reaching national and international agreement on the scale. It initiated or promoted the meetings for discussion of the differences between the L55 and 55E Scales proposed respectively by Van Dijk and Durieux, and by Clement. These were the meetings held August 26 and 27, 1955 in Leiden (before the Low Temperature Conference in Paris) [10], July 30, 31, and August 1, 1957 in Washington (before the Low Temperature Conference in Madison) [11], and June 13, 14, and 16, 1958 in Leiden (before the meeting of the Advisory Committee on Thermometry of the International Committee on Weights and Measures in Sèvres) [12]. Also, the National Bureau of Standards promoted the arrangement which sent Dr. Logan of the U.S. Naval Research Laboratory to work in the Kamerlingh Onnes Laboratory from January 22, to March 14, 1958.

The Scale agreed upon at Leiden, June 13 to 16, 1958 was presented to the Advisory Committee on Thermometry of the International Committee on Weights and Measures at its meeting in Sèvres, June 20 and 21, 1958. The recommendation of the Advisory Committee to the International Committee was as follows [12]:

“Le Comité Consultatif de Thermométrie,
“avant reconnu la nécessité d’établir dans le domaine des très basses températures une échelle de température unique,
“ayant constaté l’accord général des spécialistes dans ce domaine de la physique,
“recommande pour l’usage général l’ “Echelle ^4^He 1958,” basée sur la tension de vapeur de l’hélium, comme définie par la table annexée.
“Les valeur des températures dans cette échelle sont désignées par le symbole T_58_.”

The table of He^4^ vapor pressures that was sent to the International Committee with this recommendation was the table distributed at the Kamerlingh Onnes Conference on Low Temperature Physics at Leiden, June 23 to 28, 1958. It was published in the Proceedings of the Kamerlingh Onnes Conference [13].

On the recommendation of its Advisory Committee on Thermometry, the International Committee on Weights and Measures approved the “1958 He^4^ Scale of Temperatures” at its meeting at Sèvres, September 29 to October 3, 1958.

The table adopted by the International Committee on Weights and Measures was a table of vapor pressures at hundredth degree intervals. This table was expanded by Clement and Logan making table I of this paper with millidegree entries. Table I was inverted to give tables II and III which express *T* as a function of vapor pressures. Auxiliary tables were added including a table of the differences between the 1958 Scale and other earlier used scales. Linear interpolation is valid for all tables except at the lower temperature end of table IV.

The assistance at Leiden of H. ter Harmsel and C. van Rijn, students of Dr. H. van Dijk at the Kamerlingh Onnes Laboratory, with the computations for the defining and auxiliary tables is gratefully acknowledged.

Various members of the Cryogenics Branch of the Naval Research Laboratory at Washington assisted with numerous calculations which contributed toward the development of the present scale. This assistance, especially that of Dr. R. T. Swim, is gratefully acknowledged.

## Footnotes

5 Figures in brackets indicate the literature references on page 4.
